# Human leukocyte antigen G and miR-148a are associated with the pathogenesis of intrahepatic cholestasis of pregnancy

**DOI:** 10.3892/etm.2014.1986

**Published:** 2014-09-22

**Authors:** XIAOWEN ZHANG, LING YU, YILING DING

**Affiliations:** Department of Obstetrics and Gynecology, The Second Xiangya Hospital, Central South University, Changsha, Hunan 410011, P.R. China

**Keywords:** human leukocyte antigen G, miR-148a, intrahepatic cholestasis of pregnancy, placenta

## Abstract

Intrahepatic cholestasis of pregnancy (ICP) occurs mainly during the third trimester of gestation and is characterized by pruritus and elevated serum bile acid levels. The pathogenesis of this disease has yet to be elucidated. The nonclassical human leukocyte antigen G (HLA-G) is a trophoblast-specific molecule and is involved in the regulation of maternal immune response at the maternal-fetal interface. MicroRNAs (miRNAs) have an important role in a number of physiological and pathological processes. However, the roles of HLA-G and miRNAs in immune response in the pathogenesis of ICP have yet to be elucidated. In the present study, the expression of HLA-G and miR-148a in the placenta of patients with ICP was investigated. The mRNA and protein expression levels of HLA-G were markedly reduced in the placenta of patients with ICP compared with the levels in healthy pregnant females, and were negatively correlated with serum total bile acid (TBA) levels. It was also observed that miR-148a levels were markedly upregulated in the placenta and peripheral blood of patients with ICP. Furthermore, the mRNA and protein expression levels of HLA-G in the placenta were negatively correlated with the miR-148 levels in the placenta, but not in the peripheral blood, while the miR-148a levels in the placenta were positively correlated with serum TBA levels. These results suggest that the downregulation of HLA-G is probably caused by the upregulation of miR-148a in the placenta, and miR-148a in the placenta may contribute to the pathogenesis of ICP via the inhibition of HLA-G expression.

## Introduction

Intrahepatic cholestasis of pregnancy (ICP) is a liver disorder specific to pregnancy, characterized by maternal pruritus in the third trimester of gestation and increased levels of serum bile acid. Although the symptoms disappear quickly in mothers following delivery, the high level of bile acid is detrimental to the development of fetal organs and the functions of the umbilical cord and placenta, and may result in preterm labor and elevated prenatal mortality (1–3). The etiology of ICP is complex and has yet to be fully elucidated; however, a recent study suggested that ICP may be associated with the immunity imbalance in pregnant patients (4).

Numerous studies have investigated the mechanisms of maternal-fetal immune tolerance. The placenta functions as an immunological barrier between the mother and the fetus, which protects the fetus from maternal immune rejection (5). Human leukocyte antigen G (HLA-G) belongs to the HLA nonclassical class I heavy chain paralogues and mediates the protection from the deleterious effects of natural killer cells, cytotoxic T lymphocytes, macrophages and mononuclear cells (6–8). HLA-G is highly expressed in the placental trophoblast cells and thus has an important role in the regulation of immune tolerance in pregnancy and the maintenance of gestation (6–8). In a previous study, we demonstrated that the expression of HLA-G protein was significantly reduced in the extravillous trophoblasts of patients with ICP, whilst tumor necrosis factor-α (TNF-α) and TNF-α/interleukin-4 (IL-4) were significantly elevated in patients with ICP (9). Therefore, it may be predicted that the downregulation of HLA-G and the imbalance of T helper 1 (Th1)/Th2 cytokines may be associated with the occurrence of ICP.

MicroRNAs (miRNAs) are a class of cellular RNAs that affect the translation or stability of target messenger RNAs. Active, mature miRNAs are 17–24 bases long, and are single-stranded RNA molecules expressed in eukaryotic cells, which are highly conserved across species (10–12). miRNAs have an important role in a number of physiological and pathological processes. Numerous studies have demonstrated that miRNAs have a key role in the differentiation of immune cells and regulation of immune responses (13,14). miR-148 is an important miRNA associated with immunity, and has a role in the regulation of immune balance, the innate immune response of dendritic cells, antigen presentation and inhibition of the production of numerous inflammation-associated cytokines (15,16). It has been demonstrated that miR-148a and miR-152 directly downregulate HLA-G expression by binding to the 3′ untranslated region (UTR), and are expressed at low levels in the placenta compared with other healthy tissues (17). These findings are consistent with the specific high expression levels of HLA-G observed in the placenta. However, the expression pattern of miRNAs and their functions in the placenta of patients with ICP remain unclear.

In the present study, the expression levels of HLA-G and miR-148a in the placenta of patients with ICP and healthy subjects were investigated, as well as the levels of miRNA-18a and bile acids in the peripheral blood. The correlation between HLA-G or miR-148a expression and serum total bile acid (TBA) levels was also analyzed. This study may provide new evidence for understanding the pathogenesis of ICP.

## Materials and methods

### Patients and sample collection

Between December 2011 and January 2013, 37 patients underwent a cesarean section at the Department of Obstetrics, Second Xiangya Hospital, Central South University (Changsha, China). These included 20 patients with ICP (mean age, 28.40±4.057 years; gestational week at delivery, 37.44±0.891) and 17 healthy subjects (mean age, 28.82±4.812 years; gestational week at delivery, 37.81±0.570). The enrollment criteria for patients with ICP were as follows: pruritus and jaundice in the third trimester of pregnancy; no signs of chronic liver disease, skin disease or symptomatic cholelithiasis; elevated levels of aminotransferases and TBAs; and normal cholestasis following delivery. The healthy subjects did not have any history of gallstones or cholecystopathy, pruritus, drug consumption, hepatitis or any other diseases associated with hepatobiliary function. None of the patients in the present study received immune and hormone therapy during pregnancy.

Fresh placenta tissues were obtained during the cesarean section. The tissues were immediately frozen in liquid nitrogen and then stored at −80°C for RNA and protein isolation. Blood samples from patients with ICP were collected prior to drug treatment, while blood samples from normal pregnant women were collected following overnight fasting. The serum was obtained by centrifugation and stored at −20°C until further analysis. The serum TBA was measured using an enzymatic cycling assay. Briefly, the cells are lysed and the total protein is collected. The protein is then centrifuged for 10 min by 15,294 × g at 4°C. Bile acid is specifically oxidized by 3 alpha-hydroxy steroid dehydrogenase and Thio-NAD+, and then 3-ketosteroid and Thio-NADH are generated. In addition, with the presence of 3 alpha-hydroxy steroid dehydrogenase and NADH, the 3-ketosteroids transfer into bile acid and NAD+. This procedure is repeated multiple times to amplify the amount of bile acids, determine the absorbance of Thio-NADH generated and obtain the value of bile acid.

Informed consent was obtained from all subjects and the protocol was approved by the Ethics Committee of the Second Xiangya Hospital. All data were recorded in a computerized database.

### Quantitative polymerase chain reaction (qPCR)

Total RNA was extracted from frozen placenta tissues using TRIzol^®^ reagent (Invitrogen Life Technologies, Grand Island, NY, USA) in accordance with the manufacturer’s instructions. qPCR was performed using the SYBR^®^ Green Real-time PCR Master Mix (Toyobo, Osaka, Japan). The primers of HLA-G were as follows: forward, 5′-CCACCACCCTGTCTTTGACTAT-3′ and reverse, 5′-GTGTATCTCTGCTCCTCTCCAG-3′. GAPDH was used as a control using the following primers: forward, 5′-GCACCGTCAAGGCTGAGAAC-3′ and reverse, 5′-TGGTGAAGACGCCAGTGGA-3′. The results were analyzed using the Ct (2^−ΔCt^) method using the following formula: ΔCt = (Ct_HLA-G_ − Ct_GAPDH_).

In order to analyze miR-148a and RNU6B expression (U6 snRNA was used as a reference gene), a two-step qPCR with specific primers for miR-148a and RNU6B was performed in accordance with the manufacturer’s instructions. The primers were designed by Applied Biosystems (Foster City, CA, USA). The qPCR was performed using a PRISM 7300 Sequence Detection system (Applied Biosystems), with a 25-μl reaction system containing 10 μl PCR Master Mix (Ambion, Austin, TX, USA) and 1.33 μl reverse transcription product, and each sample was analyzed in triplicate. PCR was performed at 95°C for 10 min, followed by 40 cycles of 95°C for 15 sec and 60°C for 60 sec. The results represent three independent assays. Relative expression of miR-148a in ICP tissues was calculated using the comparative Ct (2^−ΔCt^) method, using U6 small nuclear RNA (Ambion) as the endogenous control.

### Western blot analysis

Total proteins were extracted and separated using 10% sodium dodecyl sulfate-polyacrylamide gel electrophoresis and then transferred onto polyvinylidene fluoride membranes (Millipore, Bedford, MA, USA). The membranes were incubated with anti-human HLA-G antibody (Abcam, Cambridge, MA, USA) at 4°C overnight, and then incubated with horseradish peroxidase-conjugated secondary antibody (Santa Cruz Biotechnology, Santa Cruz, CA, USA) for 1 h at room temperature prior to detection by chemiluminescence (SuperSignal West Pico Chemiluminescent Substrate; Thermo Scientific, Rockford, IL, USA). β-actin antibody (Santa Cruz Biotechnology) was used as a loading control. The intensity of bands was quantified using Quantity One software (Bio-Rad Laboratories Ltd., Hercules, CA, USA), and the relative intensity of the protein bands was determined against β-actin.

### Statistical analysis

The SPSS software package, version 13.0 (SPSS, Inc., Chicago, IL, USA) was used for statistical analysis. All data are represented as the mean ± standard error (SE). One-way analysis of variance (one-way ANOVA) was used for statistical analysis. The correlation analyses were evaluated by linear regression using GraphPad Prism 5 (GraphPad Software, Inc., La Jolla, CA, USA). P<0.05 was considered to indicate a statistically significant difference.

## Results

### HLA-G mRNA and protein are significantly reduced in the placenta in patients with ICP

**Figure 1:**
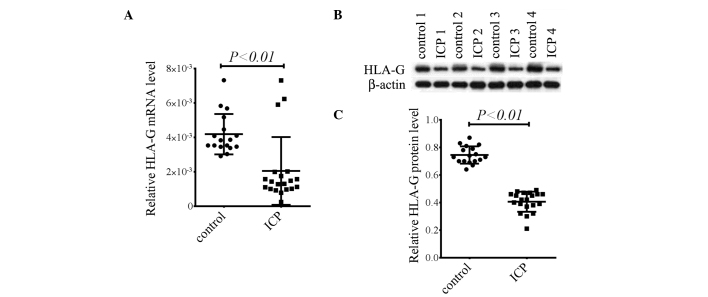
Placental HLA-G expression in patients with ICP and healthy pregnant females. (A) HLA-G mRNA expression in the placenta. The relative mRNA expression was determined using GAPDH as a reference. (B) Typical illustrations of HLA-G protein expression from a number of patients with ICP and control patients. (C) HLA-protein expression in the placenta. The relative protein expression levels were determined using β-actin as a reference. HLA-G, human leukocyte antigen G; ICP, intrahepatic cholestasis of pregnancy.

Patients with ICP were verified by determining the levels of serum TBA, alanine transaminase (ALT) and aspartate transaminase (AST; data not shown). To investigate the possible role of HLA-G in patients with ICP, the mRNA expression levels of HLA-G in the placentas of patients with ICP and healthy pregnant females were analyzed. The HLA-G mRNA expression levels in the placenta were significantly reduced in patients with ICP compared with those in normal pregnant females (P<0.01, Fig. 1A). Consistent with the mRNA levels, HLA-G protein expression levels were also markedly decreased in the placentas of patients with ICP (P<0.01, Fig. 1B and C). These results indicate HLA-G may be involved in the pathogenesis of ICP.

### HLA-G expression is negatively correlated with the serum TBA level

**Figure 2:**
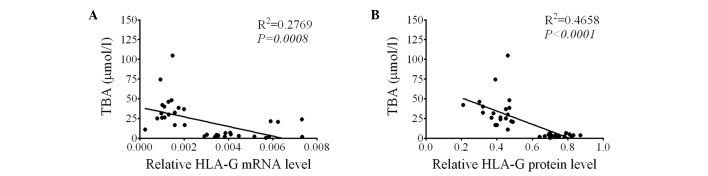
Correlation analysis between placental HLA-G and serum TBA levels. (A) The correlation between HLA-G mRNA expression levels in the placenta and serum TBA levels. (B) The correlation between HLA-G protein expression levels in the placenta and serum TBA levels. The analysis was performed by linear regression using GraphPad Prism 5 (n=37). HLA-G, human leukocyte antigen G; TBA, total bile acid.

In order to determine the association between HLA-G expression and ICP, using the data from ICP patients and healthy pregnant females, the correlation between placental HLA-G expression and serum TBA level was determined. It was observed that the HLA-G mRNA level in the placenta was negatively correlated with the serum TBA level (P=0.008, Fig. 2A). Furthermore, a negative correlation was observed between the placental HLA-G protein levels and the serum TBA level (P<0.0001, Fig. 2B).

### miR-148a levels are elevated in the placenta and serum in patients with ICP

**Figure 3:**
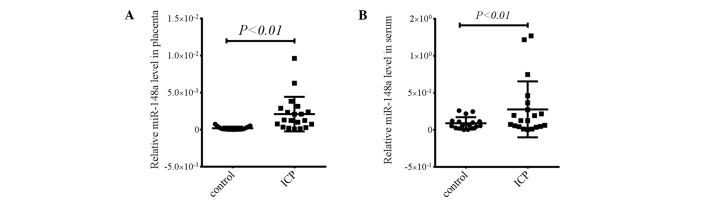
Expression of miR-148a in patients with ICP and healthy pregnant females. (A) miR-148a levels in the (A) placenta and (B) serum. Relative miR-148a expression was determined using U6 small nuclear RNA as a reference. ICP, intrahepatic cholestasis of pregnancy.

It has previously been demonstrated that miR-148a is involved in the regulation of immune response and downregulates HLA-G expression directly (16,17). Furthermore, HLA-G is highly expressed in the trophoblast cells in the placenta (6). Therefore, the present study investigated the expression of miR-148a in the placenta. The miR-148a levels in the placenta were significantly elevated in the patients with ICP compared with those in healthy pregnant females (P<0.01, Fig. 3A). In addition, miR-148a levels were measured in the serum and it was found that miR-148a levels were also upregulated in the serum of patients with ICP (P<0.01, Fig. 3B). These results suggest that the elevated miR-148a levels may inhibit HLA-G expression in the placenta of patients with ICP.

### HLA-G expression is negatively correlated with miR-148a expression levels in the placenta

**Figure 4:**
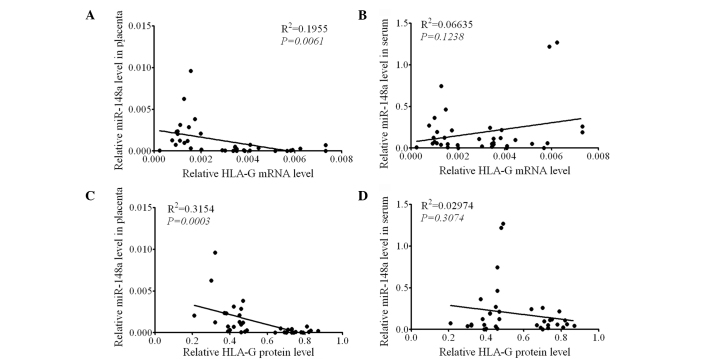
Correlation analysis between HLA-G expression levels and miR-148a levels. (A) Correlation between HLA-G mRNA expression levels in the placenta and miR-148a levels in the placenta. (B) Correlation between HLA-G mRNA in the placenta and miR-148a level in the serum. (C) Correlation between HLA-G protein in the placenta and miR-148a level in the placenta. (D) Correlation between HLA-G protein in the placenta and miR-148a level in the serum. The analysis was performed by linear regression using GraphPad Prism 5 (n=37). HLA-G, human leukocyte antigen G.

To verify the association between HLA-G expression and miR-148a levels, a correlation analysis using linear regression was performed. In all subjects it was found that the mRNA expression levels of HLA-G in the placenta were negatively correlated with the miR-148a levels in the placenta (P=0.0061, Fig. 4A), but not in the serum (P=0.1238, Fig. 4B). Furthermore, HLA-G protein expression levels in the placenta were negatively correlated with the miR-148a level in the placenta (P=0.0003, Fig. 4C), but not in the serum (P=0.3074, Fig. 4D). These results further support the hypothesis that miR-148a in the placenta is a negative regulator of HLA-G expression.

### miR-148a levels in the placenta are positively correlated with serum TBA levels

**Figure 5:**
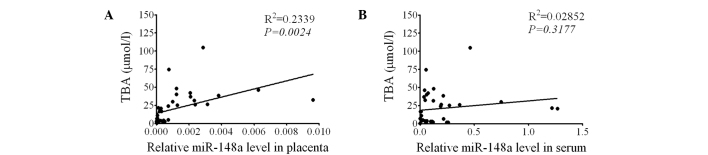
Correlation analysis between miR-148a levels and serum TBA levels. (A) Correlation between miR-148a levels in the placenta and serum TBA levels. (B) Correlation between miR-148a levels in the serum and serum TBA levels. The analysis was performed by the linear regression using GraphPad Prism 5 (n=37). TBA, total bile acid.

Since HLA-G expression was found to be negatively correlated with serum TBA levels and placental miR-148a levels, the correlation between miR-148a levels and serum TBA was then investigated. The results demonstrated that the miR-148a levels in the placenta were positively correlated with serum TBA levels (P=0.0024, Fig. 5A), while serum miR-148a was not correlated with serum TBA levels (P=0.3177, Fig. 5B). These results indicate that the miR-148a in the placenta may be involved in the regulation of TBA levels.

## Discussion

ICP is a common pregnancy-associated liver disorder, which is associated with a higher frequency of fetal distress, preterm delivery and sudden intrauterine fetal mortality (1–3). ICP is characterized by pruritus starting from the second or third trimester of pregnancy, which disappears following delivery. The most sensitive laboratory abnormality in ICP is an increase of serum TBA levels (2,3). In the present study, serum TBA levels were found to be significantly elevated in patients with ICP, which indicates that patients with ICP were correctly identified. ALT and AST are two important indices for evaluating liver cell damage (18). Although it has not yet been demonstrated that the etiology of ICP is associated with changes in ALT and AST levels, the elevated ALT and AST levels observed in patients with ICP indicate the adverse effect of ICP on liver cells.

The exact cause of ICP has yet to be elucidated; however, a number of studies have demonstrated that ICP is associated with the immunity imbalance in the placenta (4,19). The nonclassical HLA-G molecule is a trophoblast-specific molecule present in almost every pregnancy. Furthermore, HLA-G appears to be responsible for the reprogramming of local maternal immune response at the maternal-fetal interface (6,20). One study demonstrated that mixed purified HLA-G has a dual role in the first trimester placenta; low HLA-G results in an augmentation of the allocytotoxic T-lymphocyte response and induction of a Th1 cytokine response, whilst high HLA-G suppresses the allo-T-lymphocyte response and induces a Th2-type cytokine response (4). Furthermore, another study observed that the decreased expression of HLA-G in the extravillous trophoblasts of patients with ICP may cause a shift of Th1 cytokine response (21). In the present study, it was also found that the mRNA and protein expression levels of HLA-G were significantly reduced in the placentas of patients with ICP. These results are consistent with previous findings, and indicate that reduced expression of HLA-G may cause the imbalance of Th1/Th2 cytokine response and thus is associated with the pathogenesis of ICP. To verify this hypothesis, the correlation between HLA-G expression levels in the placenta and serum TBA levels was investigated in the present study. In all 37 subjects, the expression (mRNA and protein) of HLA-G in the placenta was negatively correlated with serum TBA levels, although this correlation was not observed in the ICP group or healthy pregnant females group. The reason for this difference is that the variance of HLA-G expression or serum TBA level was small in each group. Thus these data further confirm that HLA-G is involved in the pathogenesis of ICP. The downregulation of HLA-G expression in the placenta is another diagnostic marker for patients with ICP. In addition, since soluble HLA-G induces a shift in Th1/Th2 cytokine responses, HLA-G may be considered as a novel therapeutic strategy for the treatment of patients with ICP.

miRNAs are a class of small RNA molecules that regulate gene expression and are involved in a number of physiological and pathological processes, including developmental processes and immune responses. In addition, they are involved in a number of pathologies, including cancer and autoimmunity. miRNAs negatively regulate gene expression post-transcriptionally by promoting the degradation or inhibiting the translational of target mRNAs (10–14). However, to the best of our knowledge, the roles of miRNAs in the pathogenesis of ICP have not yet been investigated. In the present study it was found that the miR-148a levels were markedly elevated in the placenta and peripheral blood of patients with ICP. This suggests that miR-148a may have a role in the pathogenesis of ICP, and this is the first report regarding the potential role of miRNA in ICP. A previous study demonstrated that miR-148a negatively regulates HLA-G expression by binding to the 3′UTR (17). In the present study, it was demonstrated that the mRNA and protein expression levels of HLA-G in the placenta were negatively correlated with the miR-148a levels in the placenta but not in the peripheral blood, and this finding further confirms that miR-148a is a negative regulator of HLA-G expression. Since HLA-G expression in the placenta was negatively correlated with serum TBA levels, the association between miR-148a level and serum TBA levels was also determined. It was found that the miR-148a level in the placenta, but not in the peripheral blood, was positively correlated with serum TBA levels. This suggests that miR-148a may be associated with the changes in serum TBA levels, and HLA-G may also be involved in this association.

In conclusion, in the present study, it was found that HLA-G was reduced in the placenta of patients with ICP and was negatively correlated with serum TBA levels. In addition, it was demonstrated for the first time, to the best of our knowledge, that miR-148a is upregulated in the placenta and peripheral blood of patients with ICP. The miR-148a levels in the placenta were negatively correlated with HLA-G expression, but positively correlated with serum TBA levels. Therefore, miR-148a is a negative regulator of HLA-G expression and may regulate serum TBA levels via the inhibition of HLA-G expression. HLA-G and miR-148a may be associated with the pathogenesis of ICP; however, the exact mechanism required further investigation.
